# Blockade of vascular adhesion protein-1 attenuates choroidal neovascularization

**Published:** 2012-03-02

**Authors:** Nami Yoshikawa, Kousuke Noda, Yoko Ozawa, Kazuo Tsubota, Yukihiko Mashima, Susumu Ishida

**Affiliations:** 1 Laboratory of Retinal Cell Biology, Keio University School of Medicine, Tokyo, Japan; 2 R-Tech Ueno, Ltd., Tokyo, Japan; 3 Department of Ophthalmology, Hokkaido University Graduate School of Medicine, Sapporo, Japan; 4 Department of Ophthalmology, Keio University School of Medicine, Tokyo, Japan

## Abstract

**Purpose:**

Vascular adhesion protein (VAP)-1 is an adhesion molecule elucidated as a mediator of the leukocyte recruitment cascade. The purpose of this study was to investigate the role of VAP-1 in ocular inflammatory neovascularization using a mouse laser-induced choroidal neovascularization (CNV) model.

**Methods:**

CNV was induced with 532 nm laser irradiation in C57BL/6 mice, and production of VAP-1 protein in the retinal pigment epithelium (RPE) choroid during CNV formation was examined. CNV animals were treated with the specific VAP-1 inhibitor U-V002 or vehicle solution, and the volume of CNV tissue was evaluated with volumetric measurements. Macrophage infiltration into the CNV lesions was evaluated using two different techniques, flatmount staining and real-time polymerase chain reaction (PCR) for F4/80. The protein levels of intercellular adhesion molecule (ICAM)-1, monocyte chemoattractant protein (MCP)-1, P-selectin, and vascular endothelial growth factor (VEGF) in the RPE-choroid were measured with enzyme-linked immunosorbent assay (ELISA).

**Results:**

VAP-1 inhibition significantly suppressed CNV formation in a dose-dependent manner and reduced macrophage infiltration into CNV lesions. Furthermore, VAP-1 blockade decreased the expression of ICAM-1 and MCP-1, both of which play a pivotal role in macrophage recruitment.

**Conclusions:**

Our data suggest VAP-1 has an important role during ocular inflammatory neovascularization through leukocyte recruitment. VAP-1 inhibition may be a novel and potent therapeutic strategy in treating CNV formation.

## Introduction

Vascular adhesion protein (VAP)-1 is a dual function molecule [1], discovered in synovial endothelial cells [2]. VAP-1 is an adhesion molecule that mediates the leukocyte recruitment cascade, particularly the extravasation step [1,3], and is expressed in vascular endothelial cells throughout the body, such as those in the kidney [4], heart [5], lung [6], and ocular tissues [7]. In addition, a structural analysis revealed that VAP-1 has a high sequential homology with a group of enzymes known as semicarbazide sensitive amine oxidases (SSAOs) [8]. SSAOs catalyze the formation of inflammation-related products such as hydrogen peroxide, aldehyde, and ammonium [1]. The accumulating evidence indicates that VAP-1 is involved in inflammation via leukocyte recruitment and enzymatic reaction, thereby accounting for the impression of VAP-1 as dually functional. Recently, VAP-1 has gained attention as a biomarker and a therapeutic target for systemic inflammatory diseases [9–11].

We have reported that inhibition of VAP-1 ameliorates inflammatory changes in rat models of ocular diseases. In the endotoxin-induced uveitis (EIU) model, we showed that upon acute inflammation blockade of VAP-1 reduced leukocyte infiltration in the vitreous cavity and retina [12]. Furthermore, we demonstrated that a VAP-1 inhibitor U-V002 decreased the entrapped retinal leukocytes in the streptozotocin-induced diabetic model, a chronic vascular inflammation model induced by hyperglycemia [13]. In addition, using the rat choroidal neovascularization (CNV) model, the VAP-1 inhibitor suppressed the formation of CNV, which is a hallmark of age-related macular degeneration (AMD) and a representative type of ocular inflammatory neovascularization [14].

The objective of this study was to further investigate the involvement of VAP-1 in ocular inflammatory neovascularization using a mouse laser-induced CNV model.

## Methods

### Experimental animals and induction of choroidal neovascularization

Male C57BL/6 mice (7–8 weeks old; CLEA, Tokyo, Japan) were used. The animals were housed in plastic cages in a climate-controlled animal facility and were fed laboratory chow and water ad libitum. All animal experiments were conducted in accordance with the ARVO Statement for the Use of Animals in Ophthalmic and Vision Research and with the protocols approved by the Animal Care Committee of Keio University School of Medicine.

To generate CNV with a laser injury, mice were anesthetized with 0.2–0.3 ml of 0.5% pentobarbital sodium. Pupils were dilated with 5.0% phenylephrine and 0.8% tropicamide. CNV was induced with a 532 nm laser (Novus Spectra, Lumenis, Tokyo, Japan). Five to six laser spots (150 mW, 100 µm, 100 msec) were placed in each eye using a slit-lamp delivery system and a cover glass as a contact lens.

### Immunofluorescence microscopy

Seven days after the laser injury, the animals were perfused with PBS (136.9 mM NaCl, 2.6 mM KCl, 8.1 mM Na_2_HPO_4_•12H_2_O, 1.46 mM KH_2_PO_4_, 400 ml/kg bodyweight), and the eyes were enucleated immediately after perfusion. Frozen sections of the eyes were prepared. The sections were incubated with 10% normal goat serum blocking solution (Zymed Laboratories, San Francisco, CA) and reacted with rat monoclonal antibody against mouse VAP-1 (1:100; Abcam, Cambridge, MA). Thereafter, the sections were incubated for 2 h at room temperature with secondary antibodies (1:400, Alexa Fluor 488 goat antirat immunoglobulin G [IgG], Invitrogen, Carlsbad, CA) and mounted with mounting media with 4',6-diamidino-2-phenylindole (VECTASHIELD; Vector Laboratories, Burlingame, CA). Photomicrographs were taken with a digital high-sensitivity camera through an upright fluorescent microscope. As a negative control, the primary antibodies were replaced with normal-mouse IgG (Upstate Biotechnology, Lake Placid, NY).

### Immunoblotting

After the laser injury, the animals were sacrificed with an overdose of anesthesia, interperitoneal injection of 2ml of 5% pentobarbital sodium, at the indicated time point. The RPE-choroid tissue complex was microsurgically isolated and placed into 200 µl of lysis buffer (0.1 v/v % Triton X-100, 10 mM Tris-HCl pH 7.6, 50 mM NaCl, 29.1 mM Sodium Diphosphate Decahydrate, 47 mM Sodium Fluoride, 19.4 mM Glycerol 2-phosphate disodium salthydrate, 1 mM EDTA, 0.2 mM EGTA, pH 7.6.) supplemented with protease and phosphatase inhibitors (Sigma-Aldrich, St. Louis, MO), and then sonicated. The lysate was centrifuged (20,400x g, 15 min, 4 °C), and the supernatant was collected. Each sample containing an equal amount of total protein, quantified by NanoDrop (ND-1000; Thermo, Wilmington, DE), was separated with SDS–PAGE and electroblotted to polyvinylidene fluoride membranes (Millipore, Billerica, MA). To block the nonspecific binding, the membranes were washed with 5% skim milk and subsequently incubated with monoclonal antibody against mouse VAP-1 (1:250; BD Biosciences) or monoclonal anti-α-tubulin antibody produced in mice (1:1,000; Sigma-Aldrich) at 4 °C overnight, followed by incubation with a Peroxidase-AffiniPure Goat Anti-Mouse antibody (1:1,000; Jackson ImmunoResearch Laboratories, West Grove, PA). The signals were visualized with chemiluminescence (ECL western blotting detection reagents; GE Healthcare, Buckinghamshire, UK), according to the manufacturer’s protocol.

### VAP-1 inhibition

To block VAP-1, we used the specific VAP-1 inhibitor, U-V002, as described previously [12–14]. U-V002 is a small molecule and a derivative of 1,3-thiazole, developed and provided by R-Tech Ueno, Ltd., Tokyo, Japan. Similar to rat SSAO [12], U-V002 has a specific inhibitory property against mouse SSAO (half maximal inhibitory concentration [IC_50_], 53.1 nM), while its IC_50_ against the functionally related monoamine oxidase A and monoamine oxidase B is >10 µM. After the laser injury, the inhibitor (0.15 or 0.3 mg/kg bodyweight/day) was administered to the animals by single daily intraperitoneal injections for 7 days. The control animals received the same regimen of “the vehicle solution (1 w/v% Polysorbate 80 in Saline, pH 6.0; R-Tech Ueno, Ltd.).

### Choroidal flatmount

Seven days after laser injury and treatment with the VAP-1 inhibitor (0.15 or 0.3 mg/kg bodyweight/day) or vehicle, the size of the CNV lesions was quantified using the choroidal flatmount technique [15]. Briefly, mice were sacrificed with an overdose of anesthesia, and the eyes were immediately enucleated. The eyes were fixed in 4% PFA in PBS for 5 min. The anterior segment and retina of each eye were removed to obtain the RPE-choroidal-scleral complex, and the complex was then fixed in 4% paraformaldehyde for 2 h. The complex was then incubated with blocking solution (1% BSA, 0.5% Triton X-100, in PBS) and reacted with fluorescein isothiocyanate-conjugated isolectin B4 (1:140; Vector Laboratories, Burlingame, CA). Thereafter, the complex was mounted with mounting media (VECTASHIELD; Vector Laboratories). A scanning laser confocal microscope (FV1000; Olympus, Tokyo, Japan) with the blue argon laser wavelength (488 nm) was used to visualize CNV. Horizontal optical sections of CNV were obtained every 1 μm step from the surface to the deepest focal plane. The CNV-related fluorescence area was measured with ImageJ (USA National Institutes of Health, Bethesda, MD). The summation of the whole fluorescent area was used as the volume of CNV, as described previously [15,16].

### Quantification of macrophage infiltration

Three days after the laser injury and treatment with either the VAP-1 inhibitor or vehicle solution, the animals were sacrificed with an overdose of anesthesia, and the eyes were immediately enucleated. In the same manner as the CNV size measurement, choroidal flatmounts were prepared and incubated with rat antimouse F4/80 antigen (1:100; AbD Serotec, Oxford, UK) and goat polyclonal IgG PECAM-1 antibody (1:100; Santa Cruz Biotechnology, Paso Robles, CA). Subsequently, the tissues were mounted with mounting media (VECTASHIELD, Vector Laboratories). Photographs of the CNV lesions were taken, and the number of F4/80-positive cells was counted in a masked fashion.

### Real-time polymerase chain reaction

The expression levels of F4/80, intercellular adhesion molecule (ICAM)-1 and monocyte chemoattractant protein (MCP)-1 in the RPE-choroid complex during CNV formation were examined with real-time polymerase chain reaction (PCR). Briefly, 3 days after laser treatment the RPE-choroid tissues were obtained from eyes with or without VAP-1 inhibitor treatment and homogenized in extraction reagent (TRIzol Reagent; Invitrogen). Total RNA was prepared according to the manufacturer’s protocol. Equal amounts of total RNA extracted from the samples were reverse-transcribed with a High Capacity cDNA Reverse Transcription Kit (Applied Biosystems, Foster City, CA) at 37 °C for 1 h in a 15 μl reaction volume. Subsequently, for quantitative analysis of expression, a real-time PCR assay was performed (7500Fast; Applied Biosystems), according to the manufacturer’s protocol. Primers and TaqMan probes for mouse *F4/80*, *Icam-1*, and *Mcp-1* (Pre-Developed TaqMan Assay Reagents) were purchased from Applied Biosystems, Inc. The cycling conditions were 50 °C for 2 min, initial denaturation at 95 °C for 10 min, and 40 cycles at 95 °C for 15 s and 60 °C for 1 min. The quantity of mRNA (mRNA) expression was calculated by normalizing the C_T_ (threshold cycle) of F4/80, ICAM-1, and MCP-1 to the C_T_ of β-actin in the same sample, according to the comparative ΔΔC_T_ method.

### Enzyme-linked immunosorbent assay

Three days after the laser injury and treatment with either the VAP-1 inhibitor or vehicle solution, the animals were sacrificed by cervical dislocation, and the eyes were immediately enucleated. The RPE-choroid tissues were carefully scraped from the eyecup and placed in 200 μl of lysis buffer. The lysate was sonicated and centrifuged at 20,400x g for 15 min at 4 °C, and the ICAM-1, MCP-1, P-selectin, and vascular endothelial growth factor (VEGF) levels in the supernatant were determined with enzyme-linked immunosorbent assay (ELISA) kits for mouse ICAM-1, MCP-1, P-selectin, and VEGF (R&D Systems, Minneapolis, MN) according to the manufacturer’s protocols. The total protein concentration (Bradford technique) was determined using NanoDrop (ND-1000, Thermo).

### Statistical analysis

All results are expressed as mean±SEM with sample numbers (n) as indicated. The Student *t* test was used for statistical comparison between the groups. Differences between the means were considered statistically significant when the probability values were <0.05.

## Results

### VAP-1 expression in the choroid during choroidal neovascularization formation

**Figure 1:**
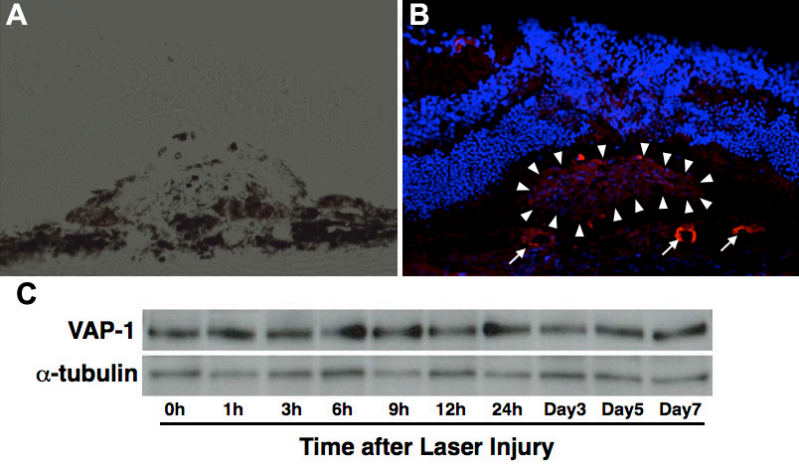
Localization and expression of VAP-1 in the choroid and CNV. **A** and **B**: Representative micrographs of a laser-induced CNV lesion. (**A**) Phase contrast image. (**B**) Fluorescent micrograph of VAP-1 (red) and cell nuclei (blue). Arrowheads and arrows indicate the localization of VAP-1 in the CNV and choroidal vessels, respectively. **C**: Immunoblotting analysis of VAP-1 and α-tubulin expression in the RPE-choroidal complex after laser injury.

To determine whether VAP-1 expression alters during CNV formation, we examined the localization of VAP-1 in CNV lesions with immunofluorescence staining and the time course of the VAP-1 protein levels with western blotting. VAP-1 was detected in endothelial cells of CNV and the choroidal vessels (Figure 1A). However, immunoblotting showed no change in the protein level of VAP-1 during CNV formation (Figure 1B).

### Impact of VAP-1 inhibition during choroidal neovascularization formation

**Figure 2:**
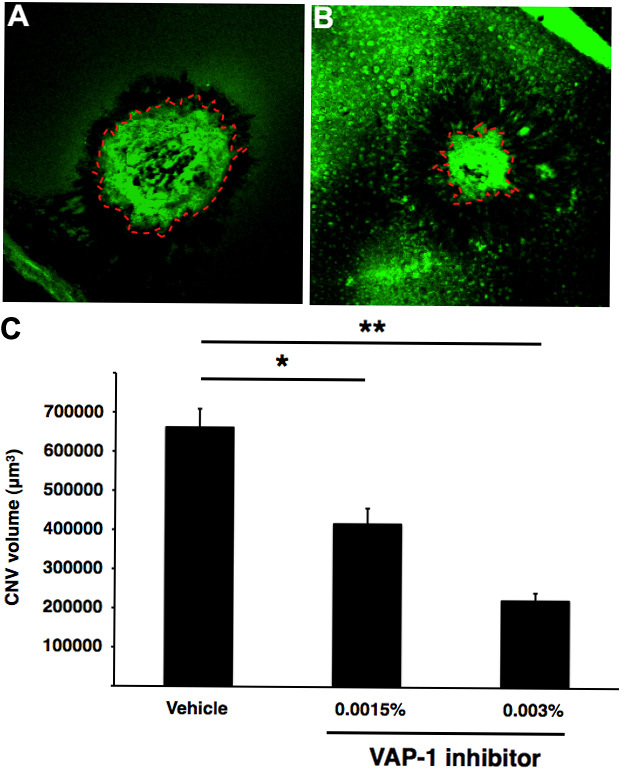
Impact of VAP-1 blockade on CNV formation. **A** and **B**: Representative micrographs of CNV lesions in the choroidal flatmounts from an animal treated with vehicle or VAP-1 inhibitor. Red dashed line shows the extent of the CNV lesions stained with FITC-conjugated isolectin B4 in flatmounted choroids. **C**: Quantitative analysis of CNV size. Bars show the average CNV size in each group. Values are mean±SEM (n=9 to 10). *, p<0.05 **, p<0.01.

To examine whether VAP-1 contributes to CNV formation in mice, we quantified the volume of the CNV in the flatmounts of the RPE-choroid complex with or without the VAP-1 blockade (Figure 2A). Seven days after the laser injury, the animals treated with the VAP-1 inhibitor (0.0015%) showed a significant decrease in their average CNV size (417189±39836 µm^3^, n=9), compared with the vehicle-treated animals (662217±47236 µm^3^, n=10, p<0.01; Figure 2B). Furthermore, a higher dose of the VAP-1 inhibitor (0.003%) reduced the CNV volume (222878±19481 µm^3^, n=8, p<0.01) even more than the animals treated with the lower dose VAP-1 inhibitor (0.0015%) (Figure 2B), indicating that the VAP-1 inhibitor suppresses CNV growth in a dose-dependent manner.

### Effect of VAP-1 blockade on macrophage influx

**Figure 3:**
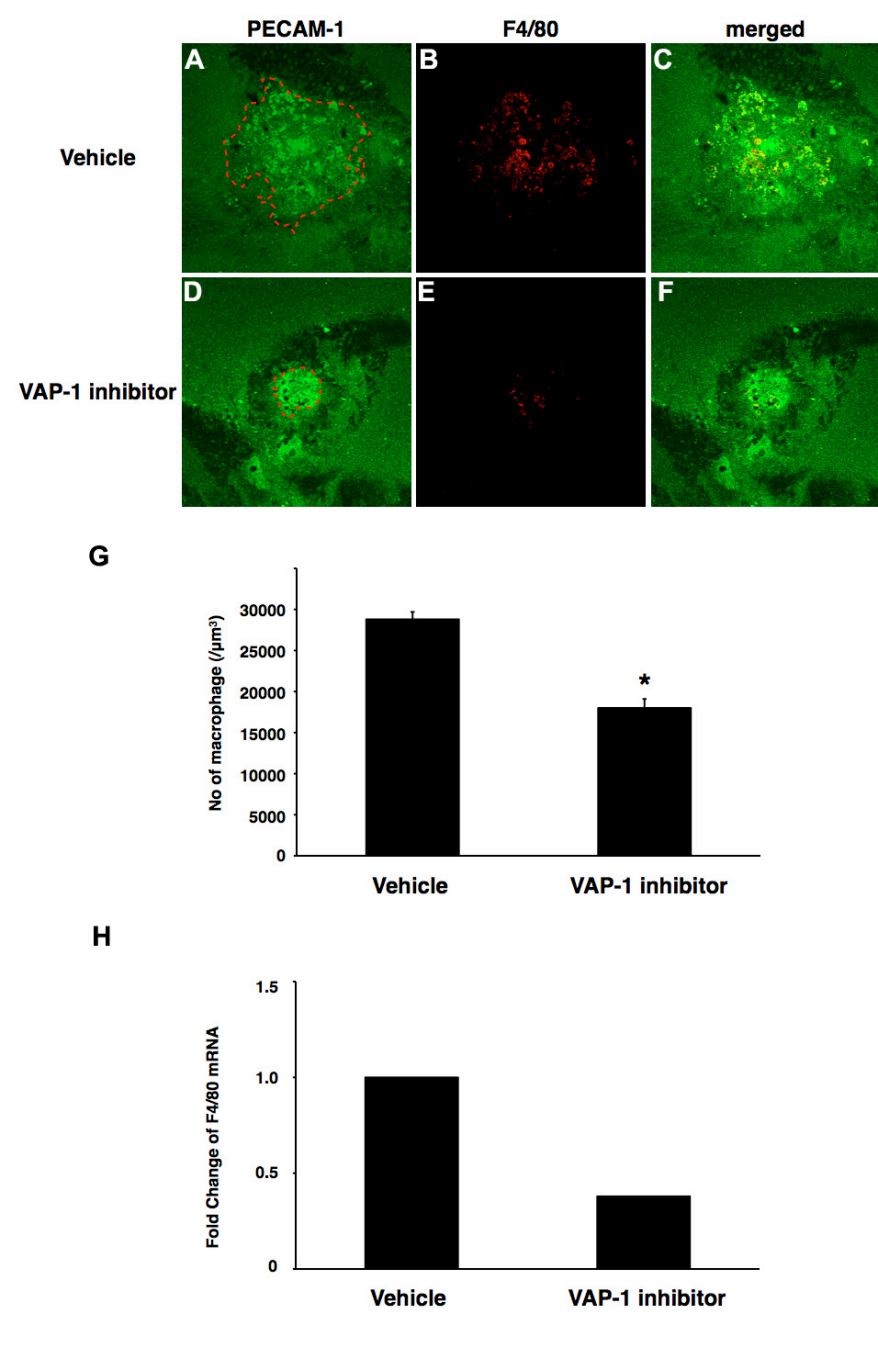
Impact of VAP-1 blockade on macrophage infiltration in CNV. **A–F**: Representative micrographs of F4/80 immunostaining in CNV lesions from an animal treated with vehicle or VAP-1 inhibitor. (Left) CNV lesions stained for PECAM-1. (Middle) Immunofluorescence staining for F4/80. (Right) Merged image. **G**: Quantitative analysis of F4/80-positive cells in CNV lesion. Bars show the average of the number of infiltrated macrophages in each group. Values are mean±SEM (n=3 to 4). *, p<0.05. **H**: Quantitative real-time PCR analysis of F4/80 expression in animals treated with vehicle or VAP-1 inhibitor (n=9 to 10).

To study whether VAP-1 inhibition reduces macrophage infiltration into the CNV lesions, we quantified the number of F4/80-positive cells in the CNV lesions of animals with and without VAP-1 inhibitor treatment (0.003%). Macrophages were recruited to the CNV lesion at 3 days after the laser injury (Figure 3A). The number of accumulated macrophages was significantly reduced by 37.6% in the animals with blockade of VAP-1 (1.80±0.11 cells/10000 (μm)^3^, n=4) compared with those with vehicle treatment (2.88±0.09 cells/10000 (μm)^3^, n=3, p<0.01, Figure 3B). Furthermore, real-time PCR showed that *F4/80* mRNA expression was downregulated by 62.1% in the animals treated with VAP-1 inhibitor (n=9) compared to that of the vehicle-treated animals (n=10, Figure 3C), in accord with the counted data of the infiltrating macrophages in the CNV lesions.

### Suppression of adhesion molecules and inflammatory molecules by VAP-1 blockade

**Figure 4:**
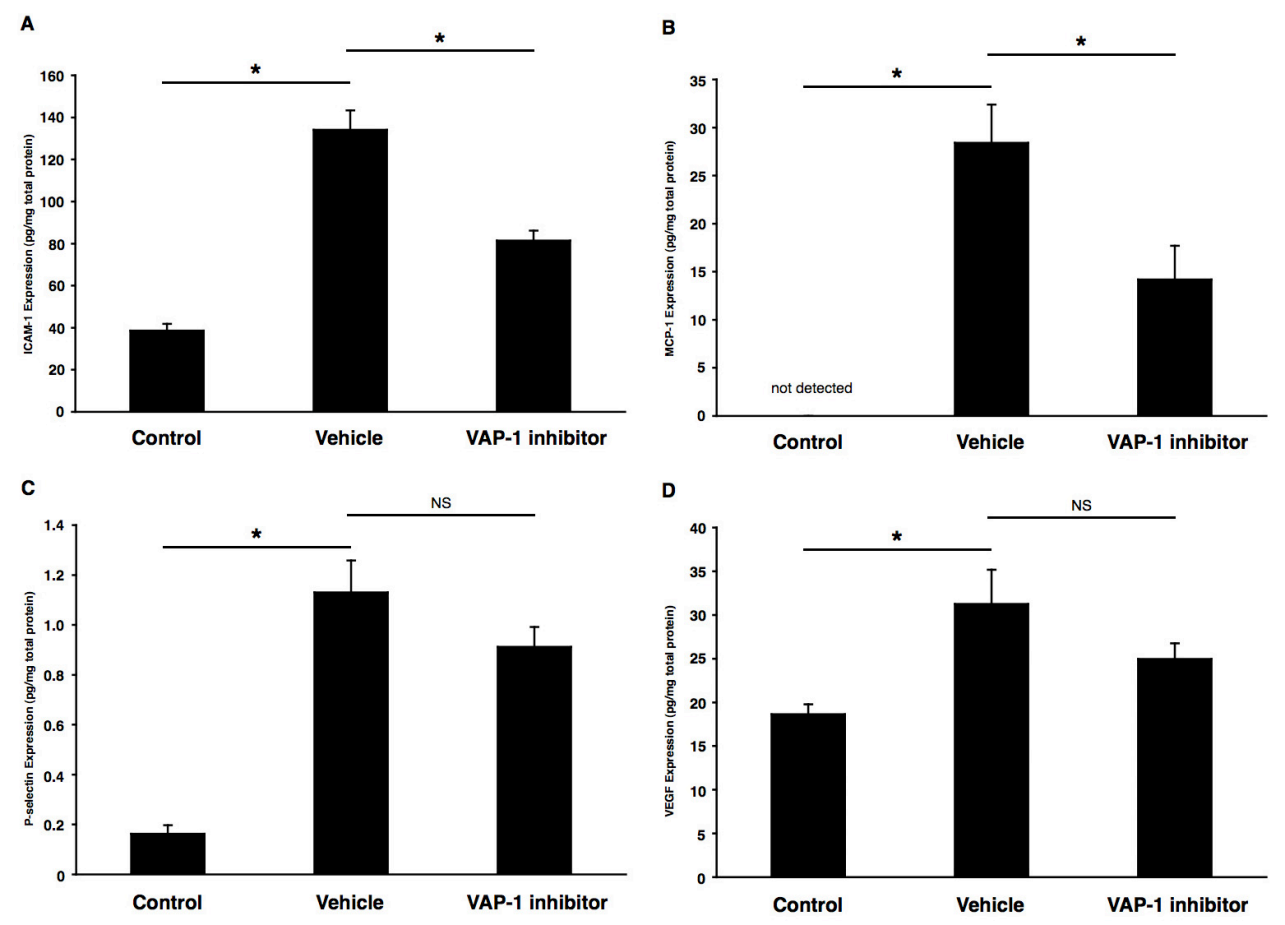
Impact of VAP-1 blockade on inflammation-associated molecules. Bars indicate the average protein levels of (**A**) ICAM-1, (**B**) MCP-1, **(C)** P-selectin, and (**D**) VEGF in the RPE-choroidal complex obtained from laser-induced CNV animals treated with vehicle or VAP-1 inhibitor at 3 days after laser photocoagulation. Values are mean±SEM (n=9 to 10). *, p<0.05.

To further explore the mechanisms by which the VAP-1 blockade suppresses CNV formation, we measured the levels of the inflammation-associated molecules, ICAM-1, MCP-1, P-selectin, and VEGF in the RPE-choroid complex with or without VAP-1 inhibition (0.003%) at 3 days after laser treatment. The ICAM-1 (38.59±3.26 ng/mg, n=10) and MCP-1 (undetectable, n=10) protein levels in the RPE-choroidal complexes of mice treated with vehicle solution were significantly increased (ICAM-1, 134.05±9.28 pg/mg, n=10, p<0.05, Figure 4A; MCP-1, 28.41±3.97 pg/mg, n=9, p<0.05, Figure 4B) at 3 days after laser injury. The ICAM-1 (81.47±4.67 pg/mg) and MCP-1 (14.19±3.50 ng/mg) protein levels were significantly reduced in the RPE-choroidal complexes of the laser-treated animals that received the inhibitor compared with the vehicle controls (Figure 4A,B). In accord with our ELISA data, real-time PCR showed that the mRNA expression levels of *Icam-1* and *Mcp-1* were downregulated by 33.4% and 11.1%, respectively, in the animals treated with VAP-1 inhibitor (n=9) compared to that of the vehicle-treated animals (n=10).

Similarly, the P-selectin (0.16±0.04 ng/mg, n=10) and VEGF (18.64±1.13 pg/mg, n=10) protein levels in the RPE-choroid complexes of normal mice were significantly lower than those in the mice with CNV (P-selectin, 1.13±0.13 pg/mg, n=10, p<0.05, Figure 4C; VEGF, 31.25±3.94 pg/mg, n=10, p<0.05, Figure 4D, respectively). However, the P-selectin (0.91±0.08 pg/mg) and VEGF (24.97±1.80 pg/mg) protein levels were not significantly reduced in the RPE-choroid complex of the animals treated with VAP-1 inhibitor (Figure 4C,D).

## Discussion

In the present study, VAP-1 inhibition suppressed the expression of MCP-1 and ICAM-1, both of which play a pivotal role in macrophage recruitment [17,18]. In addition, VAP-1 blockade decreased the number of infiltrated macrophages into the CNV lesions, which resulted in the suppression of CNV formation. Our data indicate the potential of VAP-1 as a therapeutic target in the treatment of CNV.

In accord with a previous study [14], the VAP-1 protein was detected in the CNV and choroid vessels. Since our and other groups have demonstrated that leukocyte adhesion molecules such as ICAM-1 and E-selectin were upregulated in the RPE-choroid complex with the laser-induced CNV model [14,17–19], this led us to the idea that VAP-1 was also upregulated in the CNV lesion and/or choroid during CNV formation. However, our immunoblotting study revealed that VAP-1 expression was, unexpectedly, unchanged in the RPE-choroid complex including the CNV lesions. The current data indicate that, unlike these other leukocyte adhesion molecules, VAP-1 likely is not modified in the choroid during CNV formation. Notably, VAP-1 was not increased by cytokine stimulation in cultured hepatic endothelial cells [20], and yet VAP-1 is markedly relevant to various liver diseases. The current data indicate that VAP-1 is a unique molecule that contributes to CNV formation without augmentation.

VAP-1 inhibition showed antiangiogenic effects on CNV growth in a dose-dependent manner. Thus far, extensive efforts have been focused on the therapeutic property of the VAP-1 inhibitor in systemic diseases such as stroke [21], uveitis [12], and lung injury [22], all of which arise from the inflammatory response. Similarly, inflammation underlies the pathogenesis in CNV formation [23]. Therefore, it is plausible that VAP-1 inhibition attenuates CNV formation, which is caused by chronic inflammation in human AMD. Our study elucidated that the mechanism to suppress CNV formation by VAP-1 inhibition is, at least in part, due to the reduction of macrophage infiltration into the CNV lesion. We previously demonstrated the decreased number of macrophages surrounding the CNV lesions after VAP-1 blockade using immunofluorescence staining [14], and the current data, generated by different experimental techniques able to specifically identify macrophages, *i.e.*, flatmount staining and real-time PCR for F4/80, supported the previous finding. Additionally, real-time PCR and ELISA data showed that VAP-1 blockade decreased MCP-1 expression in the RPE-choroid complex during CNV formation. In accord with our data, researchers recently reported that VAP-1 regulates monocyte recruitment to the tissues [24,25]. Furthermore, VAP-1 is involved in angiogenesis and tumor growth via controlling the migration of Gr-1+CD11b+ myeloid cells, which comprise immature macrophages and dendritic cells playing a pivotal role in tumor angiogenesis [26]. Taken together, the accumulating evidence indicates the importance of VAP-1 for angiogenesis.

The current data showed VAP-1 blockade caused the reduction of ICAM-1 in the RPE-choroid complex, whereas the data showed only a trend toward reduced P-selectin levels in the choroid with CNV. Using an animal model that manifests acute and severe ocular inflammation, the rat EIU model, we previously demonstrated that VAP-1 inhibition downregulated the expression of ICAM-1 and P-selectin in the inflamed retina [12]. In the previous study, we speculated from the data that the downregulation of ICAM-1 and P-selectin expression was due to reduced hydrogen peroxide generation through the enzymatic activity of VAP-1, the regulator for expression of the adhesion molecules [27,28]. Our data indicate that VAP-1 blockade reduces macrophage recruitment into the CNV lesion indirectly via suppression of other adhesion molecules, in addition to the blockade of VAP-1 per se.

Thus far, previous studies have demonstrated that marked suppression of VEGF is crucial for attenuation of CNV formation in the laser-induced CNV model [15,17]. However, in this study VAP-1 blockade showed weak inhibitory effects on VEGF, a key molecule for angiogenesis, whereas CNV formation was significantly suppressed. Since the data showed a trend toward reduced VEGF levels in the choroid with CNV, VAP-1 inhibition may have a weak suppressive effect on VEGF expression. Alternatively, these data may indicate that VAP-1 inhibition ameliorates ocular angiogenesis through mechanism(s) other than VEGF expression. Further evaluation is needed to elucidate the detailed mechanism(s).

In conclusion, the current data support our previous findings that VAP-1 plays a pivotal role in macrophage infiltration into the CNV lesions in the laser-induced CNV model, whereas the protein level of VAP-1 is sustained in RPE and choroidal tissue during CNV formation, indicating a unique property of VAP-1 that promotes angiogenesis without molecular upregulation. Furthermore, previous and current data show that the role of VAP-1 in CNV formation is not limited to a single animal species, but appears to be a general phenomenon of rodent biology and possibly mammalian biology. Whereas optimization and safety evaluation of the inhibitor compound are still required, this study raised expectations for the possibility of VAP-1 inhibitor as a novel and potent therapeutic strategy in the treatment of CNV formation.
